# Efficacy of Xanomeline-Trospium (KarXT) in Reducing Schizophrenia Symptoms: A Systematic Review and Meta-Analysis of Randomized Controlled Trials

**DOI:** 10.1192/j.eurpsy.2025.2199

**Published:** 2025-08-26

**Authors:** V. Astori, B. Pandolfi Arruda, B. Westphalen Pomianoski, D. Lopes Vieira, M. Prätzel Ellwanger, M. Frizzo Messinger, D. Fernandes Holanda

**Affiliations:** 1 Escola Superior de Ciências da Santa Casa de Misericórdia de Vitória, Vitória; 2 Universidade Nove de Julho, São Paulo; 3 Universidade Federal de Minas Gerais, Belo Horizonte; 4 Universidade do Contestado, Mafra; 5 Universidade Federal do Rio Grande do Sul, Porto Alegre; 6 Universidade Federal do Amazonas, Manaus, Brazil

## Abstract

**Introduction:**

Schizophrenia is a severe mental disorder often diagnosed in early adulthood, significantly impacting quality of life and increasing mortality risk. Xanomeline-trospium (KarXT), a combination of a muscarinic cholinergic receptor agonist and a peripheral antagonist, offers a potential new approach.

**Objectives:**

We aimed to perform a systematic review and meta-analysis to assess the efficacy of KarXT compared to placebo in reducing the symptoms of schizophrenia.

**Methods:**

We systematically searched PubMed, Embase, and Cochrane for randomized controlled trials (RCTs) enrolling patients with schizophrenia treated with KarXT versus placebo. Our outcomes included the overall improvement in schizophrenia symptoms, measured by the Positive and Negative Syndrome Scale (PANSS) total score, as well as specific symptom domains assessed by the PANSS positive and PANSS negative subscales. Additionally, the Clinical Global Impressions-Severity (CGI-S) score was used to measure the overall severity of the disorder. We computed mean difference (MD) with 95% confidence intervals (CIs) using R version 4.3.2. Heterogeneity was assessed using I² statistics.

**Results:**

Three RCTs were included with 640 patients, of whom 314 (49.1%) received KarXT. There were 509 males (79.5%) and 131 females (20.5%). The average body mass index (BMI) was slightly higher in the KarXT group (29.1) compared to the placebo (28.8). KarXT resulted in a greater decrease in the PANSS total score (MD: -9.74; 95% CI -12.40, -7.08; p<0.001; I²=0%; Figure 1A), PANSS positive (MD: -3.20; 95% CI -4.04, -2.36; p<0.001; I²=0%; Figure 1B), PANSS negative (MD: -1.55; 95% CI -2.28, -0.81; p<0.001; I²=22%; Figure 1C) and CGI-S score (MD: –0.60; 95% CI -0.75, -0.45; p<0.001; I²=24% Figure 2).

**Image 1:**

**Figure:**
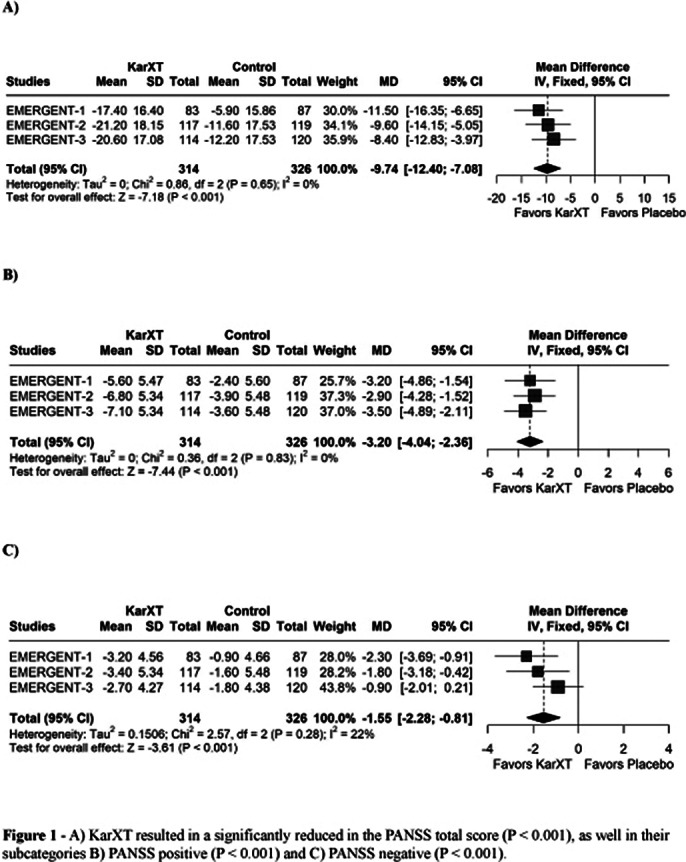

**Image 2:**

**Figure:**
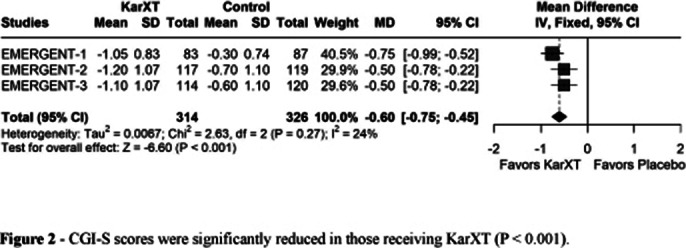

**Conclusions:**

Our systematic review and meta-analysis of RCTs showed that KarXT is significantly more effective than placebo in reducing schizophrenia symptoms.

**Disclosure of Interest:**

None Declared

